# Professional Pride During COVID-19 in a Cohort of Healthcare Workers

**DOI:** 10.3390/ijerph23030357

**Published:** 2026-03-11

**Authors:** Tanis Zadunayski, Anil Adisesh, France Labrèche, Shannon M. Ruzycki, Nicola Cherry

**Affiliations:** 1 Division of Preventive Medicine, University of Alberta, Edmonton, AB T6G 2G3, Canada; 2 Division of Occupational Medicine, University of Toronto, Toronto, ON M5S 3H2, Canada; 3 Division of Occupational and Environmental Medicine, Department of Medicine, UConn Health, Farmington, CT 06030, USA; 4 Research Department, Institut de Recherche Robert-Sauvé en Santé et en Sécurité du Travail, Montreal, QC H3A 3C2, Canada; 5 Department of Environmental and of Occupational Health, School of Public Health, Université de Montréal, Montreal, QC H3C 3J7, Canada; 6 Department of Medicine, Cumming School of Medicine, University of Calgary, Calgary, AB T2N 4Z6, Canada; 7 Department of Community Health Sciences, Cumming School of Medicine, University of Calgary, Calgary, AB T2N 4Z6, Canada

**Keywords:** COVID-19, professional pride, cohort studies, healthcare workers, mental health, personal protective equipment

## Abstract

**Highlights:**

**Public health relevance—How does this work relate to a public health issue?**

- Healthcare workers experienced both psychological strain and increased self-reported pride in their work role during the COVID-19 pandemic.
- Professional pride is an under-studied dimension of healthcare worker wellbeing with relevance for workforce sustainability during public health crises.

**Public health significance—Why is this work of significance to public health?**

- This large prospective Canadian cohort identifies individual and workplace factors associated with self-reported professional pride among healthcare workers during the pandemic.
- Findings highlight disparities in pride across job roles, care settings, and mental health status, informing targeted workforce support strategies.

**Public health implications—What are the key implications or messages for practitioners, policy makers and/or researchers in public health?**

- Professional pride may be positively associated with modifiable workplace factors such as greater support and confidence in access to protective equipment that imply care for the worker.
- Supporting mental health and acknowledging sources of professional pride may help mitigate negative experiences among healthcare workers during and after public health emergencies.

**Abstract:**

We aimed to identify circumstances associated with feelings of pride in healthcare workers (HCWs) during the COVID-19 pandemic. A prospective cohort of Canadian HCWs reported pride-reinforcing events in April 2020 (Phase 1). In spring/summer 2022 (Phase 4), they completed a self-reported retrospective comparison rating of whether they ‘now feel more [professional] pride than before the pandemic’. Among 4964 HCWs, 4360 (88%) described pride-reinforcing events in Phase 1; 3926 (79%) rated feeling more professional pride than before the pandemic in Phase 4. Teamwork (34%) and public appreciation (13%) were most cited in Phase 1. At Phase 4, male and older HCWs and community-based staff reported feeling more pride. Working as a physician, in hospital, with COVID-19 patients, and early anxiety/depression were associated with lower pride. Higher ratings were associated with greater organizational support. Many HCWs reported feeling more professional pride than before the pandemic. External support may help mitigate negative feelings.

## 1. Introduction

While many studies have highlighted the difficulties healthcare workers (HCWs) faced during the COVID-19 pandemic [1,2], focusing predominantly on stressors and their relationship with levels of depression, anxiety [3], and long-COVID [4,5], there were also notable positive experiences. These included bringing staff together [6], strengthening interpersonal work relationships [7], finding a renewed sense of purpose [8], gaining new skills [9], and feeling pride as a HCW [10,11,12].

Pride, a self-conscious positive emotion, develops from personal achievements usually from an individual’s own abilities or efforts, and involves public evaluations of oneself, typically as a socially valued person or for a valuable social outcome [13,14,15]. Feelings of pride can drive motivation and personal development [15,16], enhance job satisfaction [17], improve task performance [18], and promote perseverance [16]. While closely related to constructs such as job satisfaction and work engagement, professional pride is conceptually distinct. Job satisfaction generally reflects an evaluation of job conditions and contentment, whereas professional pride constitutes a self-conscious emotion linked to identity, moral labour, and social recognition [19]. Sociological perspectives suggest that professional pride is not merely an individual affective state, but is shaped by organizational hierarchies and the ‘hero discourse’ often invoked during crises [20,21]. It involves an interplay between one’s personal standards of performance, often termed ‘compassion satisfaction’ when related to caregiving, and the broader purpose derived from the work [12,22]. However, this sense of purpose can be threatened by ‘moral injury’ where structural constraints prevent workers from adhering to their ethical standards, potentially diminishing professional pride [23]. Furthermore, professional pride is influenced by existing occupational hierarchies and stratification within healthcare settings, which can dictate the level of symbolic recognition and resources available to different roles during a crisis [20].

Although HCWs see themselves as essential contributors to public health efforts against the pandemic [24], some HCWs did not feel appreciated and reported no pride in their work [10,12]. A Chinese study of medical students found those with anxiety or depression experienced lower pride during the pandemic [25]. Overall, it seems that professional pride during the pandemic was largely driven by a sense of contributing to society [7], teamwork [12], public appreciation [10], and personal growth [26]. Public appreciation may operate in part through “hero” narratives and symbolic recognition of HCWs [20,21]. Conversely, sustained ethical strain and perceived institutional constraint may erode professional pride through pathways of moral distress or moral injury [21,22,23]. Professional pride is increasingly recognized as an indicator of workforce engagement, job satisfaction, and retention across healthcare settings [19,27,28,29]. While professional pride has been less frequently examined in Canadian HCWs, related Canadian studies conducted during the COVID-19 pandemic have examined HCW resilience [30], coping strategies [31,32], and professional quality of life [33,34], collectively highlighting how workplace support, organizational conditions, and meaning derived from work shape HCWs’ experiences under sustained strain. As healthcare systems transition from the emergency conditions initiated by the pandemic, understanding factors that support or undermine professional pride in HCWs remains relevant for sustaining a motivated workforce during more routine operations.

Despite recognizing that many HCWs experienced pride in their roles during the pandemic, the relationship between pride and factors that influenced it remains under-explored. Few studies examined the difference in job roles, work characteristics, previous mental health status, or workplace support systems on feelings of pride during a crisis. The conceptual framework for this study was that vocational pride, as the outcome or dependent variable, was influenced by HCWs’ work experiences during the COVID-19 pandemic, including independent variables related to role and workplace conditions, such as work with patients infected with COVID-19, the provision of respiratory protection and role within the organization. Guided by this framework, we examined how these experiences were associated with reported professional pride among HCWs. The objective of this study was to identify circumstances during the COVID-19 pandemic that reinforced feelings of pride in HCW professions.

## 2. Materials and Methods

A prospective cohort of HCWs was recruited from four Canadian provinces (Alberta, British Columbia, Ontario and Quebec) in March 2020 and followed for two years [35]. HCWs included physicians (MDs) from all four provinces, registered and psychiatric nurses (RNs), licenced practical nurses (LPNs) and healthcare aides (HCAs) only from Alberta, and personal support workers (PSWs) only from Ontario.

As described in the cohort profile [35], recruitment involved a mix of strategies: professional associations and regulatory colleges disseminated invitations to their members via email and invited participation on websites; some provided direct access to their member lists. As true denominators were largely unknown, response rates to the initial invitation could not be calculated. However, 4964 HCWs were enrolled and 84% completed the Phase 4 questionnaire, demonstrating strong retention [35]. The respondents completed four online questionnaires in French or English: in April 2020 (Phase 1), in the fall of 2020 (Phase 2), in spring of 2021 (Phase 3) and in spring/summer of 2022 (Phase 4).

The baseline (Phase 1) questionnaire collected information on demographic factors (age and gender) and on pre-pandemic mental health (whether they had been treated in the 12 months prior to the pandemic for anxiety or depression) (Supplemental Material File S1A). For physicians, information on prior mental health treatment was collected at Phase 2. At Phase 1, all HCWs were asked to complete the Hospital Anxiety and Depression Scale (HADS), giving an indication of anxiety and depression in the previous week [36]. On each dimension (anxiety, depression), a total score of 11 or more can be interpreted as a clinical case [36]. Scores at Phase 1 indicating a ‘case’ of anxiety or depression were taken as a marker of mental ill-health (high anxiety or depression) early in the pandemic. Participants were asked a series of questions about their workplace, including whether they worked in a hospital, in the community, in a residential institution (such as a care-home or prison), or the patient’s own home, with each HCW recording one or more workplaces. HCWs were also asked whether they worked one-on-one with patients and whether any of these patients had been infected with COVID-19. At Phase 1, HCWs were asked to rate on a visual analogue scale their confidence in having access to all the required PPE if working with patients with COVID-19, from ‘not at all confident’ to ‘very confident’ (Q8.2, Supplemental Material File S1A) and where they were currently finding support at their workplace, on a visual analogue scale, from ‘no support at all’ to ‘very strong support’, for each source (Q8.4, Supplemental Material File S1A). These visual analogue scales were scored 0 to 100.

Members of the cohort reported the dates of their test-positive COVID-19 infections and vaccinations and were encouraged to do so proactively [37]. In three out of four provinces, public health data on infection and vaccinations were used to supplement or verify this self-reported information. In the fourth province (Ontario), only self-report data were available. A sensitivity analysis [38] demonstrated high concordance of results based on all reports and those using just validated records.

Information on the pride that participants felt in their work was collected at the start (Phase 1) and end (Phase 4) of the pandemic. At Phase 1, participants were asked to describe, using an open-text response, ‘What has been the event that has most reinforced your pride in your professional behavior?’ (Q9.2 Supplemental Material File S1A). Respondents were given the option of writing N/A (not applicable) if they preferred not to answer. A coding scheme (Supplemental Material File S2) for sources of pride was developed, tested for consistency and comprehensiveness and then applied to the responses. Coding of responses was carried out by French- or English-speaking coders, reflecting the language used by the respondent. Conflicts in the code assigned were discussed and resolved by consensus. Responses were coded as binary responses (reported or not) into 14 categories as sources of pride. Phases 2 and 3 did not include questions on pride. At Phase 4, HCWs were asked to rate whether they ‘now feel more pride as a [job role] than before the pandemic’ compared with working before the spread of COVID-19 (question J1, Supplemental Material File S1B). HCWs rated how true this statement was from ‘completely disagree’ to ‘completely agree’, with a score ranging from 0 to 100.

### 2.1. Outcome

The outcome at Phase 1 was the reporting or not of each source of pride, treated as a binary variable, coded from the open-ended question. At Phase 4, the primary outcome used was the rating of feeling more professional pride than before the pandemic (0–100) using a single-item retrospective comparison visual analogue scale.

### 2.2. Exposures

Exposures of a priori interest were job role (MD, RN, LPN, PSW, HCA), any work in a hospital setting as a binary variable (yes/no (non-hospital settings coded as ‘no’)) at Phase 1, work with patients infected with COVID-19 at Phase 1 (yes/no (no or don’t know coded as ‘no’)), history of personal COVID-19 infection (yes/no), number of inoculations against COVID-19 by Phase 4 (vaccine doses as a binary (none, some) or categorical variable (none 1–2, 3 or more)) and visual analogue ratings (0–100) of support at Phase 1 from coworkers, the work organization and the provincial heath service, and of confidence, at Phase 1, that there would be access to required PPE.

### 2.3. Potential Confounders/Effect Modifiers

Age (as a continuous variable or categorized as <40, 40 < 55, 55 years or older), gender (coded as female or not female (male or other)), mental health treatment self-reported for the 12 months before March 2020 (yes/no/unknown), and HADS anxiety/depression scores as binary variables (<11 or ≥11) were considered as potential confounders.

### 2.4. Statistical Analysis

Sources of pride reported by at least 150 HCWs at Phase 1 were tabulated by exposures and potential confounders, with the chi-square statistic used to assess the statistical significance of observed differences. The arithmetic mean ratings of greater pride at Phase 4 were compared for each categorical exposure and potential confounder, and by sources of pride at Phase 1, with differences assessed by analysis of variance (ANOVA). The relation of continuous variables (ratings of work supports and access to PPE at Phase 1) to pride at Phase 4 were assessed in linear regression models, adjusting for confounders. A multivariable linear regression model for factors associated with pride rating at Phase 4 was developed. Factors tested for inclusion were restricted to those that related to pride in a bivariate analysis (adjusting for confounders) with *p* < 0.10. They were included in the multivariable models if they contributed with *p* < 0.05: variable selection was conducted with forward and backward testing of likelihood ratios. Analyses were carried out using IBM SPSS statistical software (v29; IBM Corp., Armonk, NY, USA). Given the novel context of the pandemic and the lack of pre-existing models for pandemic-specific professional pride, variable selection was exploratory and data-driven rather than hypothesis testing-based.

### 2.5. Missing Values

Missing values for Phase 1 and Phase 4 were included in descriptive tabulations. The final analysis was restricted to those who had completed both the Phase 1 and Phase 4 questionnaires up to and beyond the pride questions. For these respondents, there were missing values on only one variable: self-reported mental ill-health in the 12 months prior to the pandemic. Even with an indicator variable for missing values, this variable did not meet inclusion criteria for the final multivariable model.

### 2.6. Ethical Approval

This study has received research ethics approval from the Health Ethics Board at the University of Alberta (Pro00099700). Unity Health Toronto Research Ethics Board also reviewed and approved the study for elements coordinated locally for participants from Ontario (REB#20-298). The research was conducted in accordance with the Declaration of Helsinki. All participants gave online written informed consent.

## 3. Results

**Figure 1:**
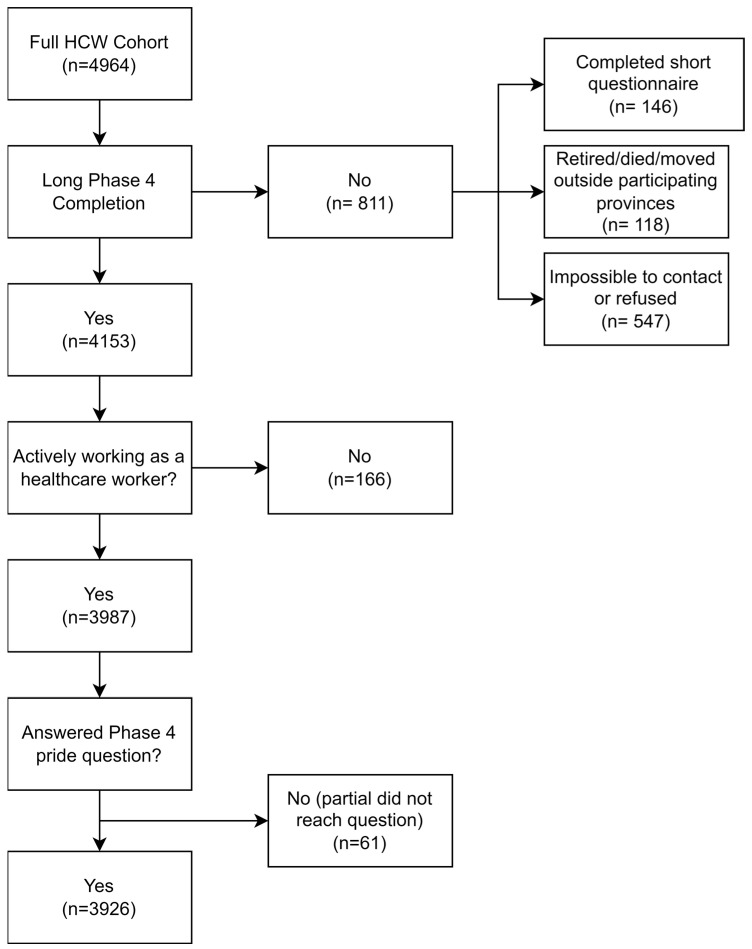
Flow chart of inclusion of HCWs in analysis for Phase 4.

Among the 4964 HCWs from the prospective cohort, 4360 completed the Phase 1 questionnaire up to and beyond the question on the event that has most reinforced your pride and 4153 (84%) completed the Phase 4 questionnaire. Of these, 3987 were actively working as a HCW at Phase 4, with 3926/4964 (79%) reporting a pride rating (Figure 1). Among these 3926, 3510 were working at Phase 1 and had completed the Phase 1 question on pride-reinforcing events.

Characteristics of participants completing the Phase 4 pride rating are shown in Table 1. The majority of HCWs were female, with a median age of 45 years (range, 19–85). Overall, the largest group was of registered nurses, followed by MDs with smaller numbers of LPNs, PSWs and HCAs. One in five (21%) participants reported being treated for anxiety or depression in the 12 months prior to the pandemic. At Phase 1, 25% (991/3926) of these participants had a high anxiety (HADS) score and 8% (321/3926) a high depression score. Over half the HCWs worked in a hospital setting, with nearly 60% (2081/3926) working with COVID-19 patients at Phase 1. Support from coworkers was, overall, rated much higher at Phase 1 than support from the employer.

Less than half (43%) of the cohort reported a positive COVID-19 infection by Phase 4, with most having only one infection. Almost all HCWs were vaccinated by Phase 4, with over 85% (3341/3926) receiving three or more doses.

Events reported as those that ‘most reinforced your pride in your professional behavior’ (Q 9.2, Supplemental Material File S1A) at Phase 1 are shown, with illustrative quotations in Supplemental Material File S2. Types of events that were reported by at least 150 HCWs were considered for further analysis (Table 2). These were: teamwork, appreciation from the public/patients, staying calm, establishing new procedures/resources, using expertise/leadership/skills, helping/supporting patients, difficult roles, adaptability and flexibility, and none (including don’t know/prefer not to answer).

Teamwork was the most reported source of pride across all professions except for PSWs, for whom staying calm was cited most often (Table 2). Appreciation from the public, and adaptability and flexibility were higher among female HCWs, whereas HCWs who did not identify as female (males and non-binary) were more likely to report no source of pride. Frequency of reports of feeling pride from teamwork and staying calm increased with age. Public appreciation had the highest reports among those aged under 40. Reports of pride in teamwork were lower (30%) in those who reported being treated for anxiety or depression before the pandemic than those who did not (35%). Those whose HADS scores suggested high anxiety or depression at Phase 1 also had lower reports for teamwork and for adaptability and flexibility. Reports of having no sources of pride was higher for those with depression. Teamwork as a source of pride was reported more often by HCWs who worked in a hospital or with COVID-19 patients. For those working at a hospital, pride in using skills was reported less frequently, and having no sources of pride was reported more often. Pride from helping patients was reported more often in those working with COVID-19 patients.

Means of Phase 4 pride ratings by demographic, mental health and workplace characteristics, infection history, and vaccination are shown in Table 3: the overall mean rating for feeling greater pride was 41.5 on a scale 0–100. Pride ratings did not differ by gender, while older workers were more likely to feel more pride in their profession than before the pandemic. PSWs had the highest mean rating of pride (45.6), and MDs the lowest (39.4). No relation was seen to a treated mood disorder before the pandemic. Those whose HADS scores suggested high anxiety or depression at Phase 1 were less likely to agree they felt more pride at Phase 4. HCWs who, at Phase 1, had worked in a hospital and those who worked one-on-one with patients reported lower Phase 4 pride ratings. Pride ratings were higher in those who were vaccinated.

The relation between sources of pride at Phase 1 and ratings of pride at Phase 4 are shown in Table 4. Higher ratings of pride at Phase 4 were found for those who reported sources of pride as adaptability and flexibility and helping patients at Phase 1. The lowest ratings of pride at Phase 4 were seen for those who did not report a source of pride at Phase 1.

The mean ratings at Phase 1 of potentially modifiable workplace factors, including workplace supports and confidence in access to PPE, are shown in Supplemental Material Table S1 (Supplemental Material File S3). In multivariable analysis, those perceiving more support from their immediate organization or provincial health service at Phase 1 reported more pride as a HCW at Phase 4. Similarly, HCWs confidence in having access to PPE at Phase 1 was related to higher ratings of pride at Phase 4.

The final multivariable model of factors influencing ratings of more pride at Phase 4 is shown in Table 5. Two initially retained variables, treatment for anxiety or depression pre-pandemic and history of a COVID-19 infection, were not retained in the final model. In this multivariable analysis, female HCWs had lower pride ratings and older HCWs higher ones. With MDs as the comparison group, PSWs, HCAs and LPNs had the highest pride ratings, whereas the ratings by RNs did not differ from that of MDs. The relation between HADS scores of high anxiety or depression at Phase 1 with lower Phase 4 pride ratings were retained, as was working in a hospital and working with COVID-19 patients. Those who received more vaccine doses gave higher pride ratings. HCWs’ pride in helping support patients at Phase 1 remained related to higher pride at Phase 4 in the multivariable model, while those who reported no source of pride at Phase 1 still showed lower Phase 4 pride ratings after adjustment for other factors. HCWs ratings of support from the immediate organization and provincial health services, along with greater confidence in access to PPE, also remained associated with higher levels of pride at Phase 4.

## 4. Discussion

This study aimed to identify circumstances that reinforced feelings of pride in HCWs during the pandemic. We found that many HCWs experienced feelings of more professional pride than before the pandemic, with PSWs and HCAs expressing the highest levels. In contrast, physicians (MDs) were less likely to agree strongly that their professional pride was higher than before the pandemic. Teamwork (34%) and public/patient appreciation (13%) were the most cited sources of pride early in the pandemic: teamwork was more frequently reported by MDs and RNs, and among those working in hospitals and with COVID-19 patients. Pride in directly helping patients early in the pandemic was a significant contributor to greater feelings of pride having worked through the pandemic (Phase 4). Older HCWs tended to have higher reports of pride than younger workers at this late point in the pandemic; female HCWs had lower reports. Anxiety and depression early in the pandemic were associated with a lower likelihood of reporting increased pride close to the end. Workplace factors also played an important role: those in hospital settings and directly working with COVID-19 patients early in the pandemic were, later, less likely to express feelings of greater pride. HCWs with greater confidence in access to PPE when working with patients with COVID-19, and those who perceived stronger support from their workplace organization and provincial health service were associated with higher pride ratings. Vaccination was also associated with enhanced pride.

A strength of this study was that data were collected prospectively, allowing analysis separating reporting of events early in the pandemic from reporting of enhanced pride two years later. Our study included HCWs from different occupations, regions and workplace settings, offering insights into professional pride across multiple healthcare contexts. The validation and correction of self-reports of infections and vaccination using provincial health records strengthened our data.

A weakness of this and other studies is uncertainty about the concept of professional pride, how to measure it and its overlap with other concepts such as resilience and the absence of stress. In this study, Phase 4 pride was assessed using a single, non-validated retrospective item comparing current feelings to the pre-pandemic state. The use of a scale without psychometric validation is a primary limitation. Further, the approach used will have been subject to unquantified recall bias and post hoc rationalization, which may have been differential. Reliance on voluntary recruitment through professional associations, potentially favouring those with higher initial professional identification, will limit the generalizability to all HCWs. Additionally, while statistically significant, the absolute differences in agreement with more pride were modest, and their practical significance uncertain: the overall mean agreement of 41.5 would suggest, assuming a score of 50 to be neutral, some unwillingness to agree with the statement of feeling more pride in groups other than PSWs and HCAs. Our analysis of open-text responses on sources of pride without supplementary inquiries to elicit the context may have led to misinterpretation by the coders. The lack of data on the sources of pride at contacts after recruitment limits our understanding of the effects on pride of changing circumstances during the pandemic.

Our findings align with earlier research which highlighted that pride often stems from directly helping patients and witnessing recoveries [39,40], from managing high workloads and stress during the pandemic through teamwork and collaborative work environments [6,12,41], and from public appreciation [10]. A longitudinal Norwegian cohort of 1996 HCWs found that HCWs felt proud yet unappreciated [12]. This discordance was also reported in a study of US pediatric nurses [8] and among public health employees [42]. Although our study did not explore directly sources that diminished pride, we noted that one in five HCWs elected not to report any source of pride early in the pandemic. Our findings remained consistent after adjusting for confounders, suggesting that observed associations were not driven only by factors unrelated to work. Although we accounted for mental health in our models, depressive symptoms may influence how participants perceive and report emotions such as pride. Depression is associated with blunted affect, which may result in underreporting positive feelings.

Similar to our findings, studies in South Korea found older nurses were more likely to experience pride [43,44,45], whereas no difference in pride by gender was seen for young medical students in China [25]. As here, mental health challenges, including anxiety and depression early in the pandemic, negatively influenced pride in these students. In our study, those working in hospitals or with patients with COVID-19 were less likely to express feelings of more pride at Phase 4. This may reflect some failure, in these settings, to cope with the extraordinary demands imposed by the pandemic, particularly in high-intensity hospital settings. It may also reflect multiple other factors in these settings, including sustained workload, ethical strain, and organizational constraints, rather than diminished professional commitment [21,22,23]. Canadian research on HCW resilience and coping during the COVID-19 pandemic similarly emphasizes how organization context, perceived support, and moral constraints shape HCWs capacity to sustain positive professional experiences under prolonged strain [30,31,32,46]. A South Korean study of hospital nurses also reported slightly lower pride in nurses who cared for COVID-19 patients [45].

Our findings regarding lower pride in hospital settings and among physicians align with theories of moral injury. High-intensity environments often exposed workers to systemic failures, where the inability to provide high-quality care may have eroded the internal reward of the profession [23,29]. Conversely, the higher pride reported by PSWs and HCAs may reflect a personal performance approach to pride, where satisfaction is derived from direct patient interaction and endurance, independent of organizational hierarchy [12], a pattern that aligns with Canadian research on professional quality of life emphasizing perceived value of work as a protective factor under stress [33,34].

Reporting of sources of pride also reflected professional role and opportunity. MDs were more likely to report being proud at baseline of establishing new resources and educating staff (13.3%) compared to PSW (6.6%), LPN (4.8%), and HCA (2.9%), underlining that all professionals do not have the same opportunity to experience all sources of pride. Research suggests that tangible safety measures and perceived organizational support can positively impact HCW engagement and coping, particularly in high-stress environments [30,47]. This is consistent with our findings that greater confidence in access to PPE, and stronger perceived support from a HCW’s workplace organization and provincial health service were associated with higher pride ratings. Multiple doses of vaccination were also associated with a greater sense of pride in our study, reflecting a prior study [48] that linked vaccination to a sense of professional responsibility and enhanced motivation.

The association between vaccination and higher pride should be interpreted with caution. All will have been offered vaccination by Phase 4 and the small number (43) not accepting it will have been non-representative. Overall, a relationship between pride and vaccination may reflect institutional alignment and a shared sense of professional responsibility. As noted by Sherman & Klinenberg [21], alignment with public health mandates can reinforce a sense of professional identity and purpose during a crisis.

Pride in profession is under-researched, and our results suggest areas where more information would be helpful, particularly about the drivers and barriers to professional pride. The lower likelihood of reporting heightened pride during the pandemic among MDs and RNs warrants further exploration. The heightened pride among PSWs at the end of the pandemic may reflect lower professional pride prior to the pandemic. This also warrants further investigation and intervention. The observed link between mental ill-health and pride highlights the opportunity for workplace interventions to improve mental health. Access to adequate PPE and organization support not only ensures physical safety but may also convey to HCWs that their wellbeing is valued, encouraging a positive workplace culture that supports pride [19,29].

Professional pride has been identified as a significant factor influencing job satisfaction and motivation among healthcare workers. Xu et al. [27] found that higher levels of professional pride among nurses were significantly associated with increased job satisfaction and motivation. Similarly, Kim and Lee [28] reported that professional pride positively influences job satisfaction and retention intentions among nurses. Although our study did not directly measure these outcomes, the observed variation in pride suggests initiatives for workforce engagement and organizational culture. Further research is warranted to explore how professional pride can be promoted and its effects on the dynamics of the healthcare workplace.

## 5. Conclusions

Overall, this study contributes to the understanding of circumstances during the pandemic that reinforced stronger professional pride in healthcare occupations. Many HCWs, particularly those that provide less specialized care, those working in the community, and those who were vaccinated, reported feeling more professional pride than before the pandemic. Prioritizing modifiable workplace factors, such as ensuring access to PPE and providing workplace support, may help to mitigate circumstances damaging to feelings of pride. Professional pride should be interpreted alongside, rather than as a substitute for, efforts to address structural and working-condition challenges faced by HCWs.

## Figures and Tables

**Table 1:** Characteristics of HCWs included in analysis who had a Phase 4 pride rating (n = 3926).

Characteristics		n	(%)
Gender	Not female	658	(16.8)
	Female	3268	(83.2)
Age, years	<40	1444	(36.8)
	40 < 55	1480	(37.7)
	55 or older	1002	(25.5)
Job role	MD	1214	(30.9)
	RN	2452	(62.5)
	LPN	60	(1.5)
	PSW	144	(3.7)
	HCA	56	(1.4)
Treated for anxiety or depression pre-pandemic	No	2749	(70.0)
	Yes	822	(20.9)
	Unknown	355	(9.0)
High anxiety ^1^ (Phase 1)	No	2570	(65.5)
	Yes	991	(25.2)
	Unknown	365	(9.3)
High depression ^1^ (Phase 1)	No	3240	(82.5)
	Yes	321	(8.2)
	Unknown	365	(9.3)
Worked in a hospital (Phase 1)	No	1656	(42.2)
	Yes	2097	(53.4)
	Unknown	173	(4.4)
Working with COVID-19 patients (Phase 1)	No	1429	(40.7)
	Yes	2081	(59.3)
	Unknown	416	(10.6)
History of COVID-19 infection (Phase 4)	No	2242	(57.1)
	Yes	1684	(42.9)
Vaccinated against COVID-19 (Phase 4)	No	43	(1.1)
	Yes	3883	(98.9)
Number of vaccine doses (Phase 4)	0	43	(1.1)
	1–2	542	(13.8)
	3 or more	3341	(85.1)
Total		3926	(100.0)
**Continuous variables (rating 0–100) ^2^**			
Currently finding support from (Phase 1)		**Mean**	**(SD)**
Coworker		76.7	22.5
Mentor		56.7	32.4
Work Organization		54.8	29.3
Provincial Health Services		47.0	29.5
Chief Medical Officer		59.5	31.4
Confidence when working with patients with COVID-19 (Phase 1) ^3^		**Mean**	**(SD)**
I have access to all the required PPE		74.4	27.3

HCA, healthcare aide; LPN, licenced practical nurse; MD, medical doctor; PSW, personal support worker; RN, registered nurse; SD, standard deviation. ^1^ HADS score ≥ 11; ^2^ excluded n = 354 (did not complete at Phase 1); ^3^ excluded n = 343 (did not complete at Phase 1).

**Table 2:** Characteristics of those reporting sources of pride in Phase 1 (n = 4360).

		Teamwork		Public Appreciation		Staying Calm		Establishing New Resources, Educating Staff		Using Expertise and Skills		Helping/ Supporting Patients		Difficult Roles, Workload, Sacrifices		Adaptability and Flexibility		None, Don’t Know, Prefer Not to Answer		Total
		n	(%)	n	(%)	n	(%)	n	(%)	n	(%)	n	(%)	n	(%)	n	(%)	n	(%)	N
Job	MD	447	(33.9)	153	(11.6)	184	(13.9)	175	(13.3)	118	(8.9)	109	(8.3)	112	(8.5)	37	(2.8)	300	(22.7)	1320
	RN	963	(35.3)	362	(13.3)	274	(10.1)	271	(9.9)	321	(11.8)	240	(8.8)	145	(5.3)	120	(4.4)	497	(18.2)	2726
	LPN	19	(30.6)	6	(9.7)	10	(16.1)	3	(4.8)	8	(12.9)	11	(17.7)	4	(6.5)	3	(4.8)	20	(32.3)	62
	PSW	21	(11.5)	23	(12.6)	33	(18.1)	12	(6.6)	15	(8.2)	13	(7.1)	7	(3.8)	5	(2.7)	75	(41.2)	182
	HCA	15	(21.4)	4	(5.7)	0	(0.0)	2	(2.9)	12	(17.1)	14	(20.0)	2	(2.9)	3	(4.3)	25	(35.7)	70
	*p* value *	<0.001		0.209		<0.001		<0.001		0.019		0.001		0.001		0.110		<0.001		
Gender	Not female	245	(32.3)	75	(9.9)	93	(12.3)	74	(9.7)	87	(11.5)	54	(7.1)	47	(6.2)	18	(2.4)	193	(25.4)	759
	Female	1220	(33.9)	473	(13.1)	408	(11.3)	389	(10.8)	387	(10.7)	333	(9.2)	223	(6.2)	150	(4.2)	724	(20.1)	3601
	*p* value *	0.396		0.014		0.469		0.392		0.565		0.060		1.00		0.020		0.001		
Age, years	<40	503	(31.0)	241	(14.8)	161	(9.9)	155	(9.6)	168	(10.4)	131	(8.1)	91	(5.6)	66	(4.1)	404	(24.9)	1623
	40 < 55	552	(34.2)	179	(11.1)	199	(12.3)	177	(11.0)	165	(10.2)	141	(8.7)	99	(6.1)	63	(3.9)	316	(19.6)	1615
	55 or older	410	(36.5)	128	(11.4)	141	(12.6)	131	(11.7)	141	(12.6)	115	(10.2)	80	(7.1)	39	(3.5)	197	(17.6)	1122
	*p* value *	0.008		0.002		0.043		0.176		0.106		0.138		0.264		0.726		<0.001		
Treated for anxiety or depression pre- pandemic ^1^	No	1148	(35.0)	413	(12.6)	361	(11.0)	361	(11.0)	368	(11.2)	278	(8.5)	191	(5.8)	122	(3.7)	671	(20.5)	3280
	Yes	306	(29.7)	130	(12.6)	136	(13.2)	99	(9.6)	101	(9.8)	106	(10.3)	79	(7.7)	46	(4.5)	222	(21.5)	1031
	*p* value *	0.002		0.988		0.055		0.203		0.200		0.076		0.033		0.283		0.457		
High anxiety ^2^ (Phase 1)	No	1075	(34.6)	378	(12.2)	341	(11.0)	346	(11.1)	343	(11.0)	283	(9.1)	181	(5.8)	132	(4.2)	651	(20.9)	3109
	Yes	390	(31.2)	170	(13.6)	160	(12.8)	117	(9.4)	131	(10.5)	104	(8.3)	89	(7.1)	36	(2.9)	266	(21.3)	1251
	*p* value *	0.031		0.197		0.088		0.085		0.590		0.407		0.109		0.034		0.812		
High depression ^2^ (Phase 1)	No	1341	(34.1)	495	(12.6)	451	(11.5)	432	(11.0)	440	(11.2)	344	(8.8)	239	(6.1)	163	(4.1)	805	(20.5)	3930
	Yes	124	(28.8)	53	(12.3)	50	(11.6)	31	(7.2)	34	(7.9)	43	(10.0)	31	(7.2)	5	(1.2)	112	(26.0)	430
	*p* value *	0.028		0.873		0.925		0.016		0.038		0.388		0.357		0.002		0.007		
Worked in a hospital (Phase 1)	No	575	(30.2)	260	(13.7)	241	(12.7)	230	(12.1)	241	(12.7)	179	(9.4)	125	(6.6)	83	(4.4)	368	(19.4)	1901
	Yes	890	(36.2)	288	(11.7)	260	(10.6)	233	(9.5)	233	(9.5)	208	(8.5)	145	(5.9)	85	(3.5)	549	(22.3)	2459
	*p* value *	<0.001		0.052		0.031		0.005		<0.001		0.270		0.356		0.122		0.017		
Working with COVID-19 patients (Phase 1)	No	560	(31.2)	226	(12.6)	227	(12.7)	234	(13.0)	196	(10.9)	140	(7.8)	98	(5.5)	71	(4.0)	382	(21.3)	1794
	Yes	905	(35.3)	322	(12.5)	274	(10.7)	229	(8.9)	278	(10.8)	247	(9.6)	172	(6.7)	97	(3.8)	535	(20.8)	2566
	*p* value *	0.005		0.962		0.044		<0.001		0.924		0.037		0.094		0.765		0.724		
Total		1465	(33.6)	548	(12.6)	501	(11.5)	463	(10.6)	474	(10.9)	387	(8.9)	270	(6.2)	168	(3.9)	917	(21.0)	4360

HCA, healthcare aide; LPN, licenced practical nurse; MD, medical doctor; PSW, personal support worker; RN, registered nurse. * Pearson Chi-square. ^1^ Excluded n = 49: 22 MD did not complete at Phase 2; 27 non-MDs did not complete at Phase 1. ^2^ HADS score ≥ 11.

**Table 3:** Mean ratings for feeling greater pride at Phase 4 (0–100) than before the pandemic (n = 3926).

		Phase 4 Pride Rating				
		Mean	(SD)	n	(%)	*p* Value *
Gender	Not female	43.0	(27.3)	658	(16.8)	0.148
	Female	41.2	(28.7)	3268	(83.2)	
Age, years	<40	36.8	(26.4)	1444	(36.8)	<0.001
	40 < 55	42.5	(29.4)	1480	(37.7)	
	55 or older	46.8	(29.0)	1002	(25.5)	
Job role	MD	39.4	(26.0)	1214	(30.9)	<0.001
	RN	40.9	(28.7)	2452	(62.5)	
	LPN	45.6	(32.7)	60	(1.5)	
	PSW	61.9	(32.6)	144	(3.7)	
	HCA	55.8	(34.5)	56	(1.4)	
Treated for anxiety or depression pre-pandemic ^1^	No	41.8	(28.1)	2749	(70.0)	0.242
	Yes	40.1	(29.2)	822	(20.9)	
High anxiety ^2,3^ (Phase 1)	No	44.0	(28.0)	2570	(65.5)	<0.001
	Yes	34.7	(28.2)	991	(25.2)	
High depression ^2,3^ (Phase 1)	No	42.3	(28.0)	3240	(82.5)	<0.001
	Yes	32.1	(30.2)	321	(8.2)	
Worked in a hospital ^2^ (Phase 1)	No	45.8	(29.4)	1656	(42.2)	<0.001
	Yes	38.3	(27.2)	2097	(53.4)	
Working with COVID-19 patients ^2^ (Phase 1)	No	44.7	(28.4)	1429	(40.7)	<0.001
	Yes	39.1	(28.1)	2081	(59.3)	
History of COVID-19 infection (Phase 4)	No	42.0	(29.1)	2242	(57.1)	0.175
	Yes	40.8	(27.6)	1684	(42.9)	
Vaccinated against COVID-19 (Phase 4)	No	26.2	(28.9)	43	(1.1)	<0.001
	Yes	41.7	(28.5)	3883	(98.9)	
Number of vaccine doses (Phase 4)	0	26.2	(28.9)	43	(1.1)	0.002
	1–2	42.3	(30.6)	254	(13.8)	
	3 or more	41.6	(28.1)	3341	(85.1)	
Total		41.5	(28.5)	3926	(100.0)	

HCA, healthcare aide; LPN, licenced practical nurse; MD, medical doctor; PSW, personal support worker; RN, registered nurse; SD, standard deviation. * ANOVA. ^1^ Excluded n = 355: 60 MDs did not complete at Phase 2; 295 non-MDs did not complete at Phase 1. ^2^ Excluded those with missing data (did not fully complete Phase 1). ^3^ HADS score ≥ 11.

**Table 4:** Relation between sources of pride (Phase 1) and rating of more pride at Phase 4 (0–100) for those who completed Phase 1 and 4 (n = 3510).

		Pride Rating at Phase 4				
Sources of Pride (Phase 1)		Mean	(SD)	n	(%)	*p* Value *
Teamwork	No	41.6	(28.5)	2274	(64.8)	0.650
	Yes	41.1	(28.1)	1236	(35.2)	
Public appreciation	No	41.4	(28.3)	3065	(87.3)	0.923
	Yes	41.3	(28.5)	445	(12.7)	
Staying calm	No	41.3	(28.3)	3099	(88.3)	0.602
	Yes	42.1	(28.6)	411	(11.7)	
Establishing new resources, educating staff	No	41.4	(28.5)	3120	(88.9)	0.962
	Yes	41.5	(27.4)	390	(11.1)	
Using expertise and skills	No	41.2	(28.4)	3126	(89.1)	0.192
	Yes	43.2	(28.2)	384	(10.9)	
Helping/supporting patients	No	41.0	(28.3)	3200	(91.2)	0.014
	Yes	45.2	(29.0)	310	(8.8)	
Difficult roles, workload, sacrifices	No	41.4	(28.4)	3279	(93.4)	0.724
	Yes	42.0	(27.8)	231	(6.6)	
Adaptability and flexibility	No	41.2	(28.4)	3371	(96.0)	0.080
	Yes	45.5	(27.7)	139	(4.0)	
None, don’t know, prefer not to answer	No	42.3	(28.4)	2824	(80.5)	<0.001
	Yes	37.8	(28.1)	686	(19.5)	
Total		41.4	(28.4)	3510	(100.0)	

SD, standard deviation. * ANOVA.

**Table 5:** Final multivariable linear regression model for ratings (0–100) of more pride at Phase 4 (for those completing Phase 1 and 4; n = 3510).

Factor		Final Model		
		β	95% CI	*p* Value *
Gender	Not female	0.00	–	–
	Female	−3.76	−6.42 to −1.10	0.006
Age	Continuous (19 to 85)	0.13	0.05 to 0.21	0.002
Job role	MD	0.00	–	–
	RN	1.15	−1.11 to 3.41	0.319
	LPN	8.69	0.95 to 16.42	0.028
	PSW	22.42	16.76 to 28.07	<0.001
	HCA	11.07	3.18 to 18.96	0.006
High anxiety ^1^	No	0.00	–	–
(Phase1)	Yes	−6.05	−8.20 to −3.90	<0.001
High depression ^1^	No	0.00	–	–
(Phase 1)	Yes	−4.82	−8.15 to −1.49	0.005
Worked in a hospital	No	0.00	–	–
(Phase 1)	Yes	−3.45	−5.45 to −1.45	<0.001
Working with COVID-19 patients (Phase 1)	No	0.00	–	–
	Yes	−3.17	−5.13 to −1.21	0.002
Number of vaccine doses (Phase 4)	Continuous (0 to 5)	3.47	1.84 to 5.10	<0.001
Pride: Helping/supporting patients	No	0.00	–	–
(Phase 1)	Yes	3.25	0.08 to 6.42	0.045
Pride: Adapting and flexibility	No	0.00	–	–
(Phase 1)	Yes	1.61	−2.97 to 6.19	0.492
Pride: None, don’t know, prefer not to	No	0.00	–	–
answer (Phase 1)	Yes	−3.45	−5.77 to −1.13	0.004
Workplace Support (Phase 1)				
Coworker Work Organization Provincial Health Services	Continuous (0 to 100)	−0.05	−0.10 to −0.01	0.031
	Continuous (0 to 100)	0.06	0.02 to 0.10	0.006
	Continuous (0 to 100)	0.13	0.09 to 0.17	<0.001
Confidence in access to PPE (Phase 1)	Continuous (0 to 100)	0.05	0.01 to 0.08	0.012
N participants		3510		

CI, confidence interval; HCA, healthcare aide; LPN, licenced practical nurse; MD, medical doctor; PPE, personal protective equipment; PSW, personal support worker; RN, registered nurse. * Multivariable linear regression. ^1^ HADS score ≥ 11.

## Data Availability

The data on which this article are based will be made available on reasonable request to the corresponding author.

## Supplementary Materials

The following supporting information can be downloaded at: https://www.mdpi.com/article/10.3390/ijerph23030357/s1, Supplemental Material File S1: Questions used for data analysis from Phase 1 (S1A) and Phase 4 (S1B) questionnaires; Supplemental Material File S2: Sources of pride coding scheme; Supplemental Material File S3: Table S1: Linear regression between modifiable workplace characteristics at Phase 1 with rating of more pride at Phase 4.

## Abbreviations

The following abbreviations are used in this manuscript:

ANOVA	Analysis of variance
HADS	Hospital Anxiety and Depression Scale
HCAs	Healthcare aides
HCWs	Healthcare workers
LPNs	Licenced practical nurses
MDs	Physicians
N/A	Not applicable
PPE	Personal protective equipment
PSWs	Personal support workers
RNs	Registered and psychiatric nurses
US	United States
