# Supplementary material for: Professional Pride During COVID-19 in a Cohort of Healthcare Workers
Source: Int J Environ Res Public Health. 2026 Mar 11;23(3):357. doi: 10.3390/ijerph23030357 (PMC13027184; doi:10.3390/ijerph23030357)
Supplement: Supplementary file 1 [file ijerph-23-00357-s001.zip › Supplemental Material File S3_IJERPH.pdf]

Professional pride during COVID-19 in a cohort of healthcare workers

Supplemental Material File S3. Table S1

Supplemental Table S1. Linear regression between modifiable workplace characteristics at phase 1 with rating of more pride at phase 4

Table S1. Linear regression between modifiable workplace characteristics at phase 1 with rating of more pride at phase 4 (for those completing phase 1 and 4; n= 3510)

	Ratings of supports/confidence at Phase 1		Coefficients of regression with pride rating at Phase 4					
			Bivariate			Multivariable*		
**a) Currently finding support from:**	**Mean**	**(SD)**	**β**	**95% CI**	**P value**	**β**	**95% CI**	**P value**
Coworker	76.8	(22.5)	0.06	0.02 to 0.10	0.005	-0.08	-0.13 to -0.03	0.001
Mentor	56.8	(32.4)	0.08	0.06 to 0.11	<0.001	0.00	-0.03 to 0.04	0.927
Work Organization	55.0	(29.2)	0.18	0.15 to 0.22	<0.001	0.10	0.05 to 0.14	<0.001
Provincial Health Services	47.1	(29.5)	0.22	0.19 to 0.25	<0.001	0.16	0.12 to 0.21	<0.001
Chief Medical Officer	59.6	(31.4)	0.14	0.11 to 0.17	<0.001	0.02	-0.02 to 0.06	0.350
**b) Confidence when working with patients with COVID 19:**	**Mean**	**(SD)**	**β**	**95% CI**	**P value**	**β**	**95% CI**	**P value**
I have access to all the required PPE	74.6	(27.2)	0.11	0.08 to 0.14	<0.001	–	–	–
**N participants**			3510			3510		

CI, confidence interval; PPE, personal protective equipment

* Multivariable model including ratings of support from coworkers, mentor, work organization, provincial health services, chief medical officer
