# Supplementary material for: Professional Pride During COVID-19 in a Cohort of Healthcare Workers
Source: Int J Environ Res Public Health. 2026 Mar 11;23(3):357. doi: 10.3390/ijerph23030357 (PMC13027184; doi:10.3390/ijerph23030357)
Supplement: Supplementary file 1 [file ijerph-23-00357-s001.zip › Supplemental Material File S1_IJERPH.pdf]

Professional pride during COVID-19 in a cohort of healthcare workers

Supplemental Material File S1. Questions used for data analysis from phase 1 and 4 questionnaires

A. Phase 1 (April 2020)
B. Phase 4 (Spring/summer 2022)

Supplemental Material File S1

Phase 1

A.1 Which gender do you identify with?

○ Male

○ Female

○ Other

A.2 What is your age in years (today)?

____

Q4.1 What role have you held while in contact with patients? (check all that apply)

☐ [job type] working in an inpatient setting in a hospital

☐ [job type] in ambulatory or outpatient settings in a hospital

☐ [job type] in an emergency room

☐ [job type] in a community health setting

☐ [job type] providing clinical support to a residential institution or contained community (such as care home, prison, first nations), please specify

____

☐ [job type] providing clinical support to a workforce, please specify

____

☐ Other [job type] role, please specify

____

Q7.0 Since March 6th has your work involved one-on-one contact with known, presumed or suspected Covid-19 patients?

- ◯ Yes
- ◯ No
- ◯ Don't know

Q8.2 Please mark on the line below **how confident** you feel about working with patients with known, presumed or suspected COVID-19.

	**Not at all confident**	**Very confident**
I have access to all the required PPE		

Q8.4 Please mark on the line below to show where you will **find support** during this time.

	No support at all Very strong support
My colleagues or co-workers	
A senior colleague or mentor	
My immediate organization	
[Provincial] Health Services	
[Provincial] Chief Medical Officer of Health	

Q9.2 What has been the event that has most reinforced your pride in your professional behaviour?
(If you prefer not to answer, please put "N/A" and go to the next question)

____

____

____

____

____

Q13.3.1 In the 12 months up to March 6th, did you receive any treatment for anxiety?

- ○ Yes (1)
- ○ No (2)

Q13.3.2 In the 12 months up to March 6th, did you receive any treatment for depression?

- ○ Yes (1)
- ○ No (2)

Phase 4

J1 Please mark on the line below **how true** the following statements are about your work, during your most recent week at work since [last questionnaire date], compared with working before the spread of COVID-19 in early March 2020

I now feel more pride as a HCA than before the pandemic	
