# Supplementary material for: Professional Pride During COVID-19 in a Cohort of Healthcare Workers
Source: Int J Environ Res Public Health. 2026 Mar 11;23(3):357. doi: 10.3390/ijerph23030357 (PMC13027184; doi:10.3390/ijerph23030357)
Supplement: Supplementary file 1 [file ijerph-23-00357-s001.zip › Supplemental Material File S2_IJERPH.pdf]

Professional pride during COVID-19 in a cohort of healthcare workers

Supplemental Material File S2

Sources of pride coding scheme used to code participant open text responses, with illustrative quotes of the nine most reported pride sources (N=4360)

Source/Event reinforcing pride	Description	Illustrative Quotes	N (%)
Teamwork	Teamwork, supporting staff and colleagues, health care solidarity, helping the team, helping the team cope with stress, pride in organization.<br>*seeing colleagues and other health care workers doing good work, acting professionally, helping.	*MD-0350, male:* “All HCW looking out for each other”<br>*RN-0208, female:* “The team I work with and their dedication and eagerness to help each other”<br>*RN-0655, female:* “working with such a great team that is so adaptable to finding solutions to problems and supportive of each other during the crisis has been amazing”	1465 (33.6)
Appreciation: Public/patients	Appreciation from public/patients.	*MD-0286, female:* “I feel grateful/proud when patients thank me for my work, which has been more frequent throughout the pandemic”<br>*PSW-0090, female:* “My clients and their show of support and gratitude. Also having the name ‘PSW’ being recognized in public because we are generally left out as being a part of the health care system. PSW is not normally mentioned anywhere”	548 (12.6)
Staying calm	Staying calm, continuing as normal even under new stresses, commitment to the job and to patients<br>*being patient	*MD-0019, female:* “That I'm remaining calm and using evidence to guide decisions, and in doing so, trying to keep the nursing staff calm as well”<br>*PSW-0011, female:* “My ability to remain calm when you are facing the unknown. Able to go to work everyday and do my job the best to my ability”	501 (11.5)
Establishing new resources, educating staff	Establishing new procedures/resources, helping educate staff, arranging for virtual care/new technologies, preparing for the future.	*MD-0099, male:* “Being able to do virtual consultation & prescriptions for suitable patients”<br>*MD-0227, female:* “Working with emergency staff to develop covid protocol quickly and effectively”	463 (10.6)

		RN-0061, female: “My pride is in how we have all gotten together to find different ways to teach students not in hospital but through technology”	
Using expertise & skills	Expertise, leadership, knowledge and skills, technical skills.	*MD-0098, female:* “leading the COVID airway team for my hospital”<br>*HCA-0007, male:* “This pandemic has taught me to practice clinical skills to the utmost to prevent spread of any infection”<br>*MD-0481, female:* “[providing] guidance and leadership to the nursing staff I am working with on how to deal with this infectious disease as I have experience from the H1N1 pandemic as well as working through many infectious disease outbreaks throughout my career”	474 (10.9)
Helping/supporting patients	Helping patients, supporting patients. *participant specifically says helping or supporting patients.	*HCA-0213, female:* “Being able to empathize and calm patient[s] when the anxiety of the whole pandemic gets to them due to the isolation and lack of visitations from friends and family”<br>*LPN-0023, female:* “I've been able to support my patients through this difficult time and providing them with tools to stay in contact with their family (eg. setting up skype and phone calls) so they still feel connected despite the visitor ban in hospitals”	387 (8.9)
Difficult Work	Voluntarily working in difficult/dangerous roles, taking on more work, making sacrifices for the public good (e.g. choosing to self-isolate when it causes a decrease in pay, coming out of retirement, choosing to redeploy to a higher stress/higher risk job/location)	*PSW-0431, female:* “The fact that we have to wear the proper PPE every day with the Patients”<br>*MD-0357, male:* “Volunteering to provide extra support and backup coverage for the hospital in preparation for a possible surge of COVID patients”	270 (6.2)
Adaptability and Flexibility	Adapting to change, flexibility.	*MD-0081, female:* “Our flexibility with non-stop process changes”<br>*RN-0313, male:* “adaptability of myself and coworkers in the many changes and stressors within the emergency environment.”	168 (3.9)
None, don’t know, N/A (prefer not to answer)	None, status quo, nothing has changed<br>I don’t know/I don’t understand the question, ?<br>Put N/A if do not want to answer, N/A	*MD-0023, male:* “There has been no part of this that has made me proud.”<br>*RN-1441, female:* “I don't think I have experienced anything that reinforced my pride”	917 (21.0)
